# Suture of Tendons

**Published:** 1854-09

**Authors:** 


Suture of Tendons.—In April last, M. Chessaignac present to the Surgical Society of Paris a young girl, who five month previously had received, by falling upon a piece of glass, a transverse wound at the anterior and inferior part of the fore-arm. This having suppurated some time, healed up; an inability to flex the thumb and indicator followed, and the patient was taken to St. Antoine Hospital. It was evident that the inferior end of the tendon adhered firmly to the cicatrix. It was necessary to replace the superior end of the tendon in opposition with the inferior; to effect this the following operation was performed:

The injured tendons were exposed by dissecting up a rectangular flap, the lower margin of the wound corresponding to the cicatrix. The tendons were intact; and the median nerves occupied their proper position. The superior extremity of the cut tendons not being in view, the dissection was carried a little higher up, when the abrupt and bulky end of a tendon was seen. This extremity was drawn in contact with the lower one, and they were kept in position by a suture, the ends of which were brought out of the wound. The flap of integument was replaced by sutures and dressings, and the hand and fore-arm were kept in a forcibly flexed position. At the end of six days union was almost complete, and the patient began to flex the indicator. In less than fifteen days the patient was discharged, having recovered the ability to flex the thumb and forefinger.—*Gazette des Hôpitaux.*

